# The Memory Trace Supporting Lose-Shift Responding Decays Rapidly after Reward Omission and Is Distinct from Other Learning Mechanisms in Rats

**DOI:** 10.1523/ENEURO.0167-16.2016

**Published:** 2016-11-17

**Authors:** Aaron J. Gruber, Rajat Thapa

**Affiliations:** Canadian Centre for Behavioral Neuroscience, University of Lethbridge, Lethbridge, AB, T1K 6T5 Canada

**Keywords:** decay, lose-switch, memory, reinforcement, WSLS

## Abstract

The propensity of animals to shift choices immediately after unexpectedly poor reinforcement outcomes is a pervasive strategy across species and tasks. We report here that the memory supporting such lose-shift responding in rats rapidly decays during the intertrial interval and persists throughout training and testing on a binary choice task, despite being a suboptimal strategy. Lose-shift responding is not positively correlated with the prevalence and temporal dependence of win-stay responding, and it is inconsistent with predictions of reinforcement learning on the task. These data provide further evidence that win-stay and lose-shift are mediated by dissociated neural mechanisms and indicate that lose-shift responding presents a potential confound for the study of choice in the many operant choice tasks with short intertrial intervals. We propose that this immediate lose-shift responding is an intrinsic feature of the brain’s choice mechanisms that is engaged as a choice reflex and works in parallel with reinforcement learning and other control mechanisms to guide action selection.

## Significance Statement

The brain appears to use several neural systems that operate in parallel to control decisions. We provide very strong evidence here that a decision system compelling rats to shift responses after bad outcomes strongly influences decisions for several seconds after reward omission, and that its properties are distinct from other decision systems, such as those compelling rats to repeat decisions leading to good outcomes. This shift system is prevalent from the first day of training, and its properties are remarkably stable over weeks of testing. We suggest that it may be an immutable choice reflex that strongly influences decisions in the seconds after reward omission to briefly augment the output of other reinforcement learning systems.

## Introduction

Animals use various strategies when choosing among responses yielding uncertain reinforcement outcomes. These strategies may be informed by the discounted sum of many past reinforcements so as to bias choice toward actions that, on average, have provided more favorable reinforcements (Herrnstein, 1961). This is embodied by reinforcement learning and other algorithms that can account for experience-dependent choice bias that evolves over many trials (Rescorla and Wagner, 1972; Barto, 1995). The evidence that mammals can use a form of reinforcement learning to solve some tasks is overwhelming (Balleine and O’Doherty, 2010; Bromberg-Martin et al., 2010a). The brain, however, has several other robust systems for learning and memory that can strongly influence actions (McDonald and White, 1995; Stote and Fanselow, 2004; Gruber and McDonald, 2012). For instance, decisions are often disproportionately influenced by the recency of reinforcements and other proximal factors, which are not fully captured by conventional reinforcement learning algorithms; such factors can be included via additional model components so as to improve the fit of these algorithms to behavioral data from rodents and humans (Ito and Doya, 2009; Rutledge et al., 2009; Skelin et al., 2014). In particular, animals performing operant tasks for appetitive outcomes tend to repeat responses that were rewarded in immediately preceding trials (win-stay), whereas they tend to shift to alternative choices if preceding responses were not rewarded (lose-shift). These choice strategies have been reported in many studies spanning a wide array of tasks and species, including humans (Frank et al., 2007; Wang et al., 2014), nonhuman primates (Mishkin et al., 1962; Schusterman, 1962; Lee et al., 2004), rats (Evenden and Robbins, 1984; Skelin et al., 2014), mice (Means and Fernandez, 1992; Amodeo et al., 2012), pigeons (Rayburn-Reeves et al., 2013), and honeybees (Komischke et al., 2002). It is important to identify the neural mechanisms of these ubiquitous strategies to improve neurobiologically grounded theories of choice behavior.

Lose-shift responding in an operant task was recently shown to be abolished by lesions of the sensorimotor striatum in rats (Skelin et al., 2014), which is unexpected because this striatal region has been predominantly associated with the gradual formation of habits that are relatively insensitive to changes in reinforcement value as revealed by devaluation procedures and maze navigation (Yin et al., 2004; Pennartz et al., 2009). Here we reveal several new dissociated properties of lose-shift and win-stay response strategies, which can account for some apparent discrepancies in findings from distinct testing paradigms. In particular, we show that the temporal dependence of choice on previous reinforcement can present a significant confound pertinent to an array of behavioral tests and can account for the involvement of the dorsolateral striatum in the present task but not in devaluation.

## Methods

### Animals

A total of 115 male Long-Evans rats (Charles River, Saint-Constant, QC, Canada, except as noted below) were used in the experiments presented here. The correlation and temporal dependence of lose-shift and win-stay responding were determined from all animals that met the performance criterion (*n* = 98; see below); these data were collected over 14 months by four different experimenters in five distinct cohorts. The subject information (number of subjects, age at time of first testing) are as follows: *n* = 17, 88 d; *n* = 19, 110 d; *n* = 16, 100 d; *n* = 46, 99 d; and *n* = 17, 126 d. One of these cohorts (*n* = 19) was additionally used for the barrier experiment described below (Fig. 3). A different cohort (*n* = 17), which was born in-house, was used to study task acquisition (Fig. 5). The protocol for the behavioral task and the testing apparatus used was the same for each experiment. All animals weighed 350–600 g at the time of testing and were pair-housed in standard clear plastic cages in a vivarium with a 12-h light/dark cycle (lights off at 7:30 *p*.m.). The animals were allowed to habituate in the facility and were handled for at least 2 min/d for 1 week before training. Behavioral training and testing were conducted during the light phase (between 8:30 a.m. and 6:00 *p*.m.). The animals were restricted to 1 h of water access per day in individual cages and had *ad libitum* access to water on weekends; body weight was maintained at >85% of pretesting weight. All animal procedures were performed in accordance with the authors’ university animal care committee’s regulations.

### Apparatus

**Figure 1.**
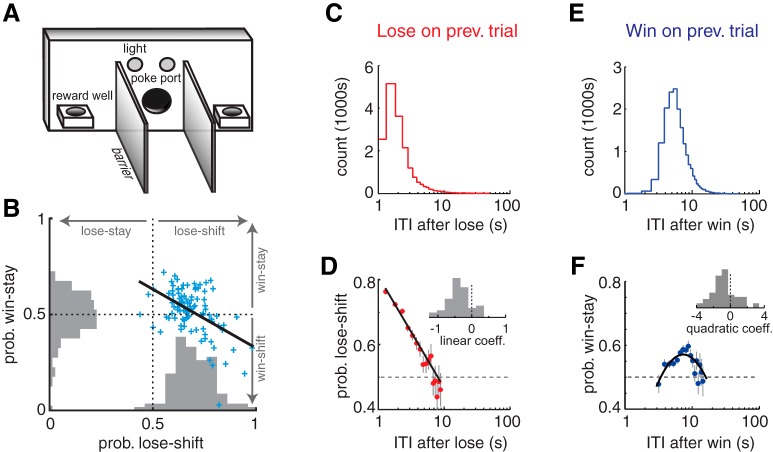
Prevalence of win-stay and lose-shift responses. ***A***, Schematic illustration of the behavioral apparatus. ***B***, Scatter plot and population histograms of win-stay and lose-shift responding, showing that these strategies are anticorrelated among subjects. ***C***, Frequency of ITIs after loss trials across the population. ***D***, Probability of lose-shift computed across the population for the bins of ITI in ***C***, revealing a marked log-linear relationship. Individual subjects also exhibit this behavior, as indicated by the nonzero mean of the frequency histogram of linear coefficient terms for fits to each subject’s responses (inset; see text for statistical treatment). ***E***, ***F***, Plots for win-stay analogous to those in ***C*** and ***D*** reveal a log-parabolic relationship with ITIs in the population and individual subjects. Vertical lines in ***D*** and ***F*** indicate SEM, and the dashed lines indicate chance levels (Prob = 0.5).

Behavioral training and testing took place in one of six identical custom-built aluminum boxes (26 × 26 cm). Each box contained two panel lights and two liquid delivery feeders on either side of a central nose-poke port (Fig. 1*A*). Infrared emitters and sensors in the feeders and central port detected animal entry. After illumination of the panel lights, a rat poked its snout into the central port to initiate a trial, and then responded by locomoting to one of the two feeders. Each feeder was equipped with an optical beam break system in the feeder to detect licking. The beam was conducted to the indentation in the feeder where the liquid reward was delivered via a pair of plastic optical fibers, and rapid changes in transmitted light intensity were detected with an industrial red/infrared (680-nm) emitter/sensor unit designed for detecting rapid interruptions in transmission while self-adjusting the emitted light power to counteract slow changes (Banner Engineering, Minneapolis, MN, model D12DAB6FP). This system sometimes registered the entry of liquid into the feeder, and could sometimes count a break as two events (the on and the off phases) because of the self-adjusting feature. The number of detected licks may therefore be biased. These biases are invariant over time, and we randomized the assignment of subjects to testing boxes each session; the biases therefore do not present significant obstacles for interpreting the relative changes in licking behavior. A 13-cm-long aluminum barrier orthogonal to the wall separated each feeder from the central port. This added a choice cost and reduced choice bias originating from body orientation. A longer 20-cm barrier was used in some sessions to increase the intertrial interval (ITI) between reward feeder exit and the subsequent nose-poke to begin the next trial. Control of the behavioral task was automated with a microcontroller (Arduino Mega) receiving commands via serial communication from custom software on a host computer. The hardware connections from the microcontroller to the sensors, valves, and lights were made via optically isolated solid-state relays (Crydom, IDC5, ODC5). We attempted to reduce acoustic startle from sounds outside of the testing chamber by presenting constant background audio stimuli (local radio station).

### Behavioral task

Individual trials of the task began with illumination of two panel-mounted lights mounted proximally to the nose-poke port and inactivation of the overhead house light. Animals then had 15 s to commit a nose-poke into the central port and subsequently respond to one of the two possible feeders. If the rat failed to respond, the apparatus would briefly enter an error state, in which the house light would illuminate and the panel lights would extinguish. The state of the lights was then reset (house light off; panel lights on) with a delay of 100–500 ms, which depended on the communication latency of the microcontroller with the host computer. The computer selected *a priori* which feeder to reward. If the rat selected this rewarded feeder, it received a 60-μl drop of 10% sucrose solution (a win) with a short delay determined by the hardware (typically <50 ms) and the fluid dynamics of the solution in the delivery system. The state of the lights did not change. If the rat chose the nonrewarded feeder, it was left empty (lose) and the apparatus would switch to the error state (house light on; panel lights off) for 100–500 ms until the system reset. This brief change in lighting was intended to signal that reward was not forthcoming. This delay was shorter than the time required for rats to locomote from the feeder to the nose-poke port when barriers are present, and so did not implement a timeout penalty. We therefore consider the task to be self-paced within the 15-s limit on trial duration. The computer implemented a “competitive” algorithm similar to previous studies (Lee et al., 2004; Skelin et al., 2014). Briefly, the algorithm examined the entire choice and reward history in the present session to exploit predictable responses and minimize the number of rewards delivered. This was done by using sequences of the most recent (the previous four) choices and reinforcements as a pattern to determine the probability of choosing each feeder based on the entire previous choice history within the current session. If the algorithm detected that either feeder was chosen more than chance in this context from all previous trials in the current session (probability >0.5 by the binomial test, *p* < 0.05), it was selected to be unrewarded for that trial. The competitive algorithm, therefore, punished predictable response patterns. The optimal solution for the rat was to be as stochastic as possible in feeder choice. Daily sessions of the task were 45 min in duration, and rats were randomly assigned a starting time and testing box for each session.

All animals were trained on the competitive choice task by gradually introducing components of the task. Initially, there were no barriers between the central port and feeders, and 50% of responses were rewarded. Subsequent sessions used the competitive algorithm. The barrier separating the nose-poke port and feeders was increased in discrete lengths (4, 8, and 13 cm) over several sessions (typically four to five). Training was complete when animals performed >150 trials with the 13-cm barrier within the 45-min session over two consecutive days. Training terminated for any subject that had not met the criterion by at least 2 d after 50% of the other members of the cohort had met the criterion. Animals typically completed training on sessions 8–11. Note that the termination of training and the inclusion criterion of 100 trial/session were implemented in attempt to homogenize experience across cohorts. Although some rats are slow to acquire the task, less than 5% fail to acquire the task with additional training. The training and inclusion criteria do not likely bias the subjects strongly toward select phenotypes. We modified the training schedule for one cohort (noted in Results) by limiting trials to 150 trials per day so that acquisition across sessions would be more homogeneous among subjects.

### Analysis

Data included up to two sessions per rat; sessions were included only if rats performed at least 100 trials (*n* = 98 rats). Population means were computed from means for each subject computed across all sessions (one point from each subject). Data were analyzed and plotted with custom-written code and built-in function of Matlab 2015a (Mathworks, Natick, MA), with the exception of ANOVA using a within-subjects design (a.k.a. RM-ANOVA), which was conducted with IBM SPSS V21 (IBM Canada, Markham, ON, Canada). We report the number of trials computed as the sum total number of complete trials within a session. We limited analyses related to reward dependence to trials in which the rat sampled only one reward feeder between trials. This was done to eliminate any effect of visiting the second feeder (the one not initially chosen) before the next trial. The probability of lose-shift was calculated as the probability that the subject would shift feeder choice in trials after reward omission. Likewise, the probability of win-stay was calculated as the probability that the subject would repeat the selection of feeders on trials immediately after rewarded trials. In defining consecutive trials, we include only trials that were <20 s apart.

We used the Matlab function “fitnlm” for fitting function parameters to the relationship between response switching probability and ITIs. Fits were weighted by the number of samples used to compute probabilities to minimize the effect of variance in the data points derived from low numbers of samples. We quantitatively validated the model fits to data collected under different conditions by computing the difference in the area under the curve (AUC) for each animal in each condition. We chose constant-sized bins in linear time that had sufficient samples in all conditions (*n* > 25) to compute probabilities. Invariance of conditions should thus result in no change in the AUC. Some subjects did not have sufficient samples in each ITI bin for each condition (e.g., non-overlapping ITI distributions due to motoric slowing) and were excluded from the AUC analysis. We also used the models of the ITI–probability relationships to estimate the motoric effects of treatments, which otherwise present confounds of the treatments on response probabilities. To do this, we used the Matlab function “predict” to predict the expected change in the probability of lose-shift for each animal according to its change in the base 10 logarithm of ITIs across conditions (barrier) using the model.

We compared the predictive power of a standard reinforcement learning algorithm called Q-learning (Watkins and Dayan, 1992) with that of win-stay/lose-shift. We first fitted the parameters of the Q-learning algorithm independently to best fit each animal’s responses on one session as described previously (Skelin et al., 2014). We then used these parameters on the same data to compute the predicted most likely next response for each subject. Note that we are using the same data for testing and training, which yields the best possible accuracy of the model. The predictions for the win-stay/lose-shift algorithm were simply determined by the reinforcement and choice on the previous trial using the same sessions as for the Q-learning model and required no parameter fitting. We then computed the prediction accuracy for each model as the percentage of correct predictions.

The power of statistical tests was computed with SPSS for ANOVA or the software package G*Power (http://www.gpower.hhu.de/en.html) for other analyses (see Table 1). Superscript letters listed with *p*-values correspond to the statistical tests shown in Table 1.

**Table 1.** Details of statistical treatments.

**Line**	**Hypothesis (H0)**	**Test**	**df**	**Test statistic value**	**Probability**	**Outcome**	**Power of outcome**	**Sample type**	**Subjects excluded (*n*)**	**Reason for exclusion**
a	Mean of lose-shift probability across the population is not equal to 0.5.	*t*	97	19.2	1.00E–34	Reject H0	1	Subjects	0	
b	Mean of win-stay probability across the population is not equal to 0.5.	*t*	97	1.4	0.17	Accept H0	0.74	Subjects	0	
c	Relationship between win-stay and lose-shift across subjects is not linearly correlated.	Linear regression	97	32.2	1.00E–06	Reject H0	0.72	Subjects	0	
d	Relationship between lose-shift probability and ITI computed from binned aggregate data from all subjects is explained by a constant model.	*F* vs. constant model	14	398	1.00E–11	Reject H0	1	Binned probabilities	0	
e	Mean regression slope computed from the independent log-linear regression of lose-shift to ITI is not different from 0.	*t*	54	40	1.00E–40	Reject H0	1	Subjects	42	Insufficient samples for regression (criterion is ≥25 samples in 4 consecutive bins, after removing trials that follow entry of the non-chosen feeder)
f	Relationship between win-stay probability and ITI for binned data across subjects is explained by a constant model.	*F* vs. constant model	14	12.8	1.00E–03	Reject H0	0.99	Binned probabilities	0	
g	Mean regression factor for the quadratic term computed from the independent regression of lose-shift to log10(ITI) is not different from 0.	*t*	63	6.6	1.00E–08	Reject H0	0.96	Subjects	32	Insufficient samples for regression (criterion is ≥25 samples in 4 consecutive bins, after removing trials that follow entry of the non-chosen feeder)
h	Relationship between the ITI after wins and the ITI after losses is explained by a constant model.	*F* vs. constant model	97	225	1.00E–26	Reject H0	1	Subjects	0	
i	Relationship between subject-wise lose-shift probability and logarithm of the ITI after losses is explained by a constant model.	*F* vs. constant model	97	20.6	2.00E–05	Reject H0	0.99	Subjects	0	
j	Relationship between subject-wise win-stay probability and logarithm of the ITI after wins is explained by a constant model.	*F* vs. constant model	97	1.8	0.18	Accept H0	0.6	Subjects	0	
k	Response time is invariant to the trial position within sessions, independent of barrier length (i.e., main effect).	RM-ANOVA	9,864	2.8	0.003	Reject H0	0.96	Binned trials and subjects	0	
l	Anticipatory licking is invariant to the trial position within sessions, independent of barrier length (i.e., main effect).	RM-ANOVA	9,864	8.8	1.00E–06	Reject H0	1	Binned trials and subjects	0	
m	Relationship between the within-session change in anticipatory licking and total licks (per trial) is explained by a constant model.	*F* vs. constant model	8	38.7	3.00E–04	Reject H0	0.99	Binned trials	0	
n	The prevalence of lose-shift responding is invariant to the trial position within sessions, independent of barrier length (i.e., main effect).	RM-ANOVA	9,864	2.2	0.02	Reject H0	0.89	Binned trials and subjects	0	
o	Relationship between the within-session change in lose-shift prevalence and anticipatory licking is explained by a constant model.	*F* vs. constant model	8	27.8	7.00E–04	Reject H0	0.99	Binned trials	0	
*p*	ITI after loss is invariant to the trial position within sessions, independent of barrier length (i.e., main effect).	RM-ANOVA	9,864	29	1.00E–06	Reject H0	1	Binned trials and subjects	0	
q	Relationship between the within-session change in lose-shift prevalence and log ITI after loss is explained by a constant model.	*F* vs. constant model	8	24.8	1.00E–03	Reject H0	0.99	Binned trials	0	
r	Mean running speed in the presence of shorter barriers is not different from the mean running speed in the presence of the longer barriers.	*t*	18	0.05	0.96	Accept H0	0.96	Subjects	0	
s	Mean % change in A.U.C for lose-shift vs. log(ITI) due to increasing barrier length for each subject is not different from 0	*t*	16	0.09	0.93	Accept H0	0.95	Subjects (within)	2	Insufficient samples for regression (criterion is ≥25 samples in 4 bins)
t	Mean % change in A.U.C for win-stay vs. log(ITI) due to increasing barrier length for each subject is not different from 0	*t*	14	0.55	0.59	Accept H0	0.87	Subjects (within)	5	Insufficient samples for regression (criterion is ≥25 samples in 4 bins)
u	Mean change in lose-shift probability across subjects when the longer barrier is introduced is not different from 0.	*t*	18	4.7	2.00E–04	Reject H0	0.71	Subjects (within)	0	
v	Mean difference between predicted and actual lose-shift decrease due to increased barrier length is not different from 0.	*t*	18	0.14	0.89	Accept H0	0.95	Subjects (within)	0	
w	Mean change in rewarded trials due to barrier length is not different from 0.	*t*	18	2.45	0.02	Reject H0	0.92	Subjects (within)	0	
x	The prevalence of lose-shift responding is invariant to the trial position within sessions, independent of barrier length (i.e., main effect).	RM-ANOVA	6,109	1.6	0.16	Accept H0	0.42	Binned trials and subjects	0	
y	The ITI after loss is invariant to the trial position within sessions, independent of barrier length (i.e., main effect).	RM-ANOVA	6,109	5.7	3.00E–05	Reject H0	0.99	Binned trials and subjects	0	
z	Anticipatory licking is invariant to the trial position within sessions, independent of barrier length (i.e., main effect).	RM-ANOVA	6,109	6.8	4.00E–06	Reject H0	1	Binned trials and subjects	0	
aa	The prevalence of lose-shift responding is invariant to barrier length, independent of changes due to trial position in the session (i.e., main effect).	RM-ANOVA	1,18	8.3	0.01	Reject H0	0.78	Binned trials and subjects	0	
ab	The ITI after loss is invariant to barrier length, independent of changes due to trial position in the session (i.e., main effect).	RM-ANOVA	1,18	28	5.00E–05	Reject H0	1	Binned trials and subjects	0	
ac	Anticipatory licking is invariant to barrier length, independent of changes due to trial position in the session (i.e., main effect).	RM-ANOVA	1,18	0.5	0.52	Accept H0	0.9	Binned trials and subjects	0	
ad	Relationship between lose-shift responding and anticipatory licking is explained by a constant model.	*F* vs. constant model	5	10.1	0.02	Reject H0	0.58	Binned trials	0	
ae	Mean difference in win-stay probability across subjects computed after a previous win vs. two previous wins at the same feeder is not greater than 0.	*t*	48	10.2	1.00E–13	Reject H0	1	Subjects (within)	2	Insufficient occurrence of win-stay-wins sequences (criterion is ≥25)
af	Mean difference in lose-shift probability across subjects computed after a previous loss vs. two previous losses at the same feeder is not greater than 0.	*t*	32	2.2	0.99	Accept H0	1	Subjects (within)	18	Insufficient occurrence of lose-stay-lose sequences (criterion is ≥25)
ag	Mean prediction accuracy of the Q-learning model and win-stay-lose-shift is not different from 0.	*t*	34	5.2	1.00E–05	Reject H0	0.96	Subjects	0	
ah	The median probability of lose-shift on the second training session is not different from chance (0.5).	Wilcox	17		0.03	Reject H0	0.77	Subjects	0	
ai	Mean probability of lose-shift did not change across training or testing days.	RM-ANOVA	15,150	0.54	0.91	Accept H0	1	Subjects, sessions	0	
aj	Mean probability of win-stay did not change across training or testing days.	Wilcox	17		0.01	Reject H0	0.83	Subjects	0	
ak	Mean probability of win-stay did not change across training or testing days.	RM-ANOVA	15,150	2.3	5.00E–03	Reject H0	1	Subjects, sessions	0	

## Results

### Lose-shift and win-stay responding are uncorrelated and have distinct time dependences

We trained Long Evans rats to perform a noncued binary choice task in which they entered a nose-poke port to initiate a trial and then locomoted to one of two liquid sucrose feeders on either side of a barrier (Fig. 1*A*). A computer algorithm computed which feeder was to be rewarded on each trial and attempted to minimize the number of rewards delivered by first using the reward and choice history of the rat to predict its next feeder choice, and then selecting the alternate feeder to be rewarded (Lee et al., 2004; Skelin et al., 2014). The optimal strategy is a random choice on each trial; win-stay, lose-shift, or other predictable response are suboptimal on this task and result in a rate of reinforcement less than the expected maximum of 50%. Deviation from a random strategy reveals features about the brain’s learning, memory, and choice mechanisms. We examined 44,898 trials from 98 rats run in five different cohorts over 14 months. The rats in our sample performed well on the task; they collected reward on a mean of 46.0 ± 4.3% of trials (range: 41.7–55.3%) compared with an expected maximum of 50%. This is in line with the performance of nonhuman primates (47–48%; Lee et al., 2004) and rats (42 ± 1.4%; Tervo et al., 2014) on similar tasks.

We next examined how delivery (win) or omission (lose) of reward affected rats’ choice on the subsequent trial of the task. The population showed very robust lose-shift responding (68.8 ± 1.0% of trials; *t* test that mean is 50%: *t*(97) = 19.2, *p* = 1E–34^a^), but not win-stay responding (51.6 ± 11.9% of trials; *t* test, *t*(97) = 1.4, *p* = 0.17^b^). These strategies were negatively correlated among subjects (*r* ^2^ < 0.25, *F*(97) = 32.2, *p* = 1E–6^c^; Fig. 1*B*). In other words, nearly every subject showed lose-shift responding, and the more likely they were to shift after losses, the less likely they were to stay after wins. We next investigated how the effect of reinforcement on subsequent choice depends on time. Trials of the task were self-paced, and we computed ITIs as the time between the first exit of the reward feeder and the next entry into the poke port. This is the minimum amount of time that reward information, or its effect on choice, needs to be represented to affect the subsequent response. ITIs were longer after win trials than after loss trials (Fig. 1*C*, *E*), which is qualitatively consistent with postreinforcement pauses (Felton and Lyon, 1966) and the frustrative effects of reward omission (Amsel, 1958) long observed in other tasks in which animals receive reward on only a fraction of responses. The temporal effects here are much shorter than past studies, and other reported pauses seem to depend on prospective motoric requirements rather than past actions (Derenne and Flannery, 2007); it is therefore difficult to compare this aspect of our data to previous studies that have largely omitted the type of barriers we have used. As we show later, the longer ITIs after rewarded trials very likely involve the time spent licking and consuming the reward.

The effect of the reinforcement type (win/lose) on subsequent choice has a distinct dependence on ITIs. The probability of lose-shift responding has a prominent log-linear relationship with the ITI at the population level (*r* ^2^ = 0.96, df = 14; *F* statistic vs. constant model = 389, *p* = 1E–11^d^; Fig. 1*D*), suggesting exponential decay of the influence of reward omission on subsequent choice in linear time. The probability of lose-shift reaches chance level (*p* = 0.5) within 7 s for the population. This could arise either because of a within-animal process (e.g., decaying memory trace) or because of individual differences among the population (e.g., faster rats have a stronger tendency to shift). To distinguish among these possibilities, we tested whether the relationship between ITI and lose-shift was evident within subjects. Indeed, this negatively sloped log-linear relationship does fit the behavior of most individual subjects (t test that slope of fit for individual subjects was equal to 0: *t*(54) = 40.0, *p* = 1E–40^e^; Fig. 1*D* inset). This indicates that the temporal dependence occurs within individual subjects rather than exclusively at a population level.

In contrast to the log-linear temporal dependence of lose-shift, the probability of win-stay shows a log-parabolic relationship with ITI, in which it first increases to a peak at ∼8 s before decreasing (*r* ^2^ = 0.60, df = 14, *F* statistic vs. constant model = 12.8, *p* = 1E–3^f^; Fig. 1*F*). This log-parabolic relationship also fit the behavior of most individual subjects (*t* test that the distribution of quadratic coefficients fit to each subject has a mean of 0: *t*(63) = 6.6, *p* = 1E–8^g^; Fig. 1*F* inset), again indicating a within-subject effect. In sum, the choices of most individual subjects in our large sample show dependence on the time interval since the last reinforcement, consistent with a temporally evolving neural process. Moreover, the distinct temporal profiles of lose-shift and win-stay responding support the hypothesis that they are mediated by distinct neural processes. The temporal dependencies of these response types have inverse slopes near the mean ITI after wins or losses, which thereby suggests an explanation for the negative correlation between the probability of win-stay and lose-shift. If the ITI after wins and losses are correlated within animals, then faster animals will show strong lose-shift and weak win-stay, whereas slower animals will have weaker lose-shift and stronger win-stay. Indeed, the ITI after wins is highly correlated with the ITI after losses (*r* ^2^ = 0.70, *F*(97) = 225, *p* = 1E–26^h^), and the correlation between subject-wise mean lose-shift and the logarithm of the mean ITI after loss is moderately strong (*r* ^2^ = 0.17, *F*(97) = 20.6, *p* = 2E–5^i^). However, the correlation between mean log ITI after wins and win-stay among subjects is weak (*r* ^2^ = 0.03, *F*(97) = 1.8, *p* = 0.18^j^), likely because of the nonlinear dependence of win-stay on log ITI (Fig. 1*F*) and because of between-subject variance in the acquisition of win-stay as described later. In sum, the inverse relationship between lose-shift and win-stay responding among subjects (Fig. 1*B*) can likely be attributed to subject-wise variation in ITI.

**Figure 2.**
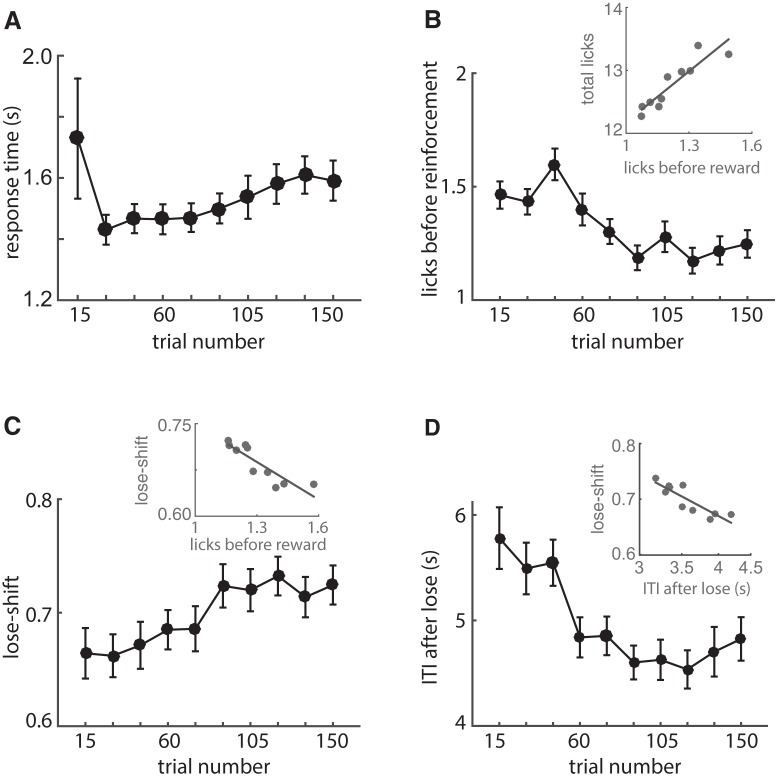
Within-session changes of dependent variables. ***A***, Mean response time (from nose-poke to feeder) over 15 consecutive trials and all animals in Fig. 1. Response time increases throughout the session after trial 30, suggesting a progressive decrease in motivation. ***B***, Mean number of licks before reinforcement, which decreases within the session. The number of these anticipatory licks correlates strongly with the total number of licks at each feeder within the session (inset). ***C***, Mean probability of lose-shift, which increases within the session and negatively correlates with licking (inset). ***D***, Mean ITI after loss trials decreases within session. The within-session variance of lose shift correlates strongly with the log of the within-session ITI after losses (inset). Error bars indicate SEM.

We next tested how motivation may affect the prevalence of lose-shift responding by quantifying the variation of dependent variables within sessions. We computed means of variables over bins of 15 consecutive trials for each animal before generating population statistics. We presume that reasonable behavioral correlates of motivation in this task are the response time (from poke-port to feeder) and the number of licks made before reinforcement time (either reward delivery or panel lights extinguishing). As rats accumulate rewards within the session, we presume their motivation decreases, and thereby expect increased response time and decreased anticipatory licking. Indeed, response time increases after the first 15 trials (RM-ANOVA main effect trial: *F*(9,864) = 2.8, *p* = 0.003^k^; Fig. 2*A*), whereas anticipatory licking decreases (RM-ANOVA: *F*(9,864) = 8.8, *p* < 1E–6^l^; Fig. 2*B*). Furthermore, anticipatory licking correlates very strongly with the total number of licks on each trial (*r* ^2^ = 0.83, *F*(8) = 38.7, *p* = 3E–4^m^; Fig. 2*B* inset), further supporting the notion that this metric reflects motivation. The decrease of licking within session contrasts the increase of lose-shift responding within sessions (RM-ANOVA: *F*(9,864) = 2.2, *p* = 0.02^n^; Fig. 2*C*). Indeed, these are strongly, and negatively, correlated (*r* ^2^ = 0.78, *F*(8) = 27.8, *p* = 7E–4^o^; Fig. 2*C* inset). This suggests that lose-shift responding is not driven by motivation. On the other hand, the ITI after losses decreases as sessions progress (RM-ANOVA: *F*(9,864) = 29, *p* < 1E–6^p^; Fig. 2*D*), and this decrease (in log space) is correlated with increased lose-shift (*r* ^2^ = 0.76, *F*(8) = 24.8, *p* = 1E–3^q^; Fig. 2*D* inset). In sum, the movement speed to the feeders and anticipatory licking decrease within sessions; these changes likely reflect decreasing motivation during the session. On the other hand, the ITI after losses decreases, likely because in part of reduced time spent licking in the feeders. Thus, the fact that lose-shift responding increases as sessions progress suggest it is more likely directly related to changes in ITI than is motivation, in agreement with the overwhelmingly strong correlational evidence of this relationship at the population and individual levels (Fig. 1*D*). Of course, motivation almost certainly plays a role in modulating the ITI, and can thereby exert indirect effects on lose-shift responding.

### Change in lose-shift responding is predicted by change in ITI: evidence for a decaying memory trace

**Figure 3.**
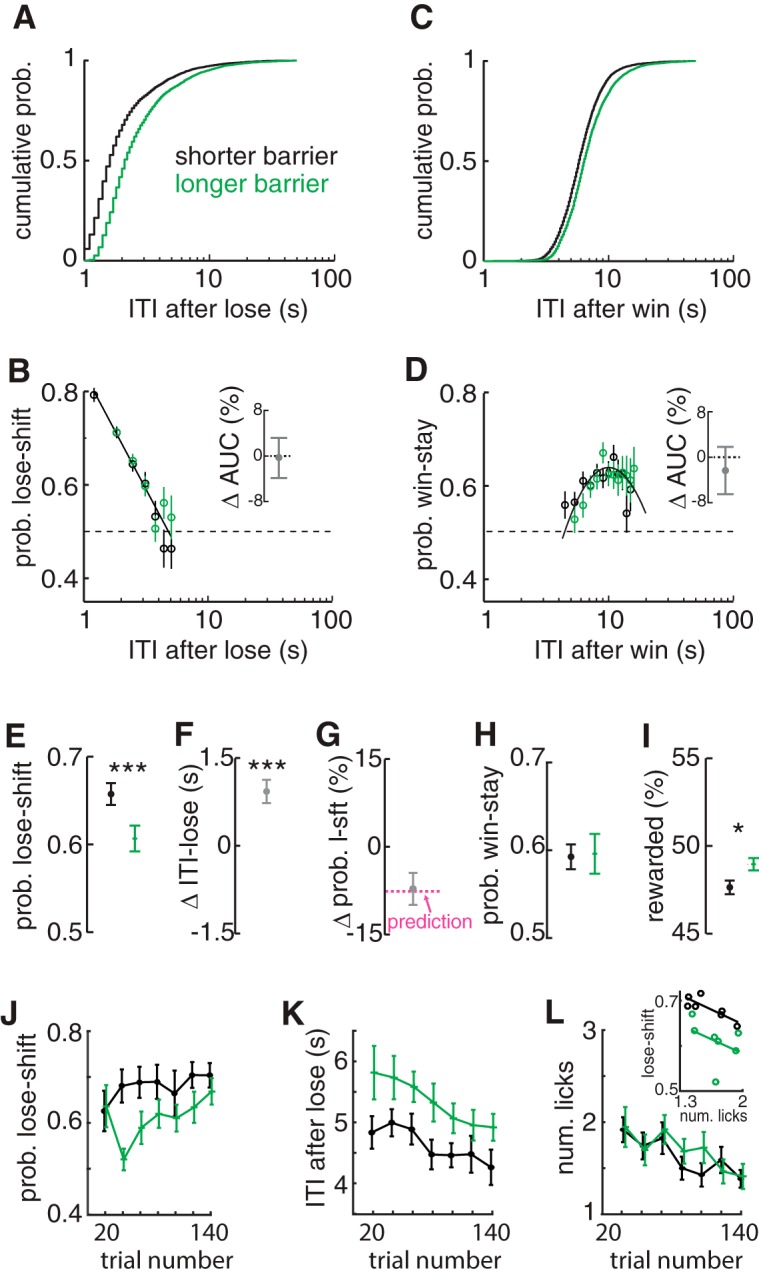
Invariance of lose-shift and win-stay models to movement times. ***A***, Frequency of population ITIs after losses showing that intervals were increased for long (green) compared with short (dark) barriers. ***B***, Probability of lose-shift computed across the population independently for short (dark) and long (green) barriers. Both conditions were fitted well by the common model (dark solid line). The change in the area under the curve computed independently for each subject between conditions shows no difference (inset), indicating that the mnemonic process underlying lose-shift responding is invariant to the ITI distribution. ***C***, ***D***, Plots of ITI and probability of stay responses after wins, showing that win-stay is also invariant to barrier length. ***E***, Mean lose-shift responding across subjects is decreased by longer barriers. ***F***, Within-subject ITI increases after loss trials under long barriers compared with short barriers. ***G***, Mean within-subject change in the probability of lose-shift due to longer barriers is predicted (magenta dashed line) by the change in ITI based on the log-linear model. ***H***, Mean probability of win-stay computed across animals is not altered by barrier length. ***I***, Long barriers led to more rewarded trials per session because of the reduction in predictable lose-shift responding. ***J***, Mean probability of lose-shift for bins of 20 trials and rats for long and short barriers, showing an increase across sessions for either barrier length. ***K***, Mean ITI after loss for each barrier condition, showing a decrease within the session. ***L***, Mean number of licks prior to reinforcement across the session, showing a decrease within sessions but no effect of barrier length. (***L***, inset) Plots of lose-shift and licking for each barrier condition, showing that licking is not sufficient to account for variance in lose-shift between barrier conditions. Statistically significant difference among group means: *p < 0.05, ***p < 0.001. Error bars show SEM.

The regression analysis of individual subjects’ responses indicates that the decrease in lose-shift responding that occurs as ITIs get longer is observed within most subjects. We hypothesize that this could reflect a decaying memory trace, analogous to decay or accumulating interference of short-term memory of other information (Mizumori et al., 1987; Altmann and Gray, 2002). The previous correlation analysis is not sufficient to rule out alternate hypotheses, such as a population component to the phenomenon. For instance, rats with short ITIs may be more sensitive to reinforcement omission than rats moving more slowly. We thus tested these hypotheses by assessing whether the dependence of choice on previous reinforcement is altered by inducing longer ITIs. The choice–ITI curve should translate (shift) to the right with increased median ITI if the choice–ITI relationship is due to a population effect, but should remain invariant to increasing ITIs if the relationship is due to a decay of a memory trace. We assessed this by alternating between short (13 cm) and long (20 cm) barriers on successive days for one cohort (*n* = 19 rats, six sessions). Rats presumably have similar motivation (i.e., thirst) regardless of the barrier length. This is supported by the fact that the decrease in the number of trials (24.0%) is proportional to the increase in median ITI (25.6%) in the session with longer barriers, suggesting that the decrease in trials is due to increased locomotion time rather than decreased motivation to complete trials. Furthermore, the running velocity during responses (nose-poke to feeder) is not affected by the barrier length (19.0 cm/s for shorter and 19.1 cm/s for longer barrier; paired *t* test of different means: *t*(18) = 0.05, *p* = 0.96^r^). Last, the amount of anticipatory licking in feeders is not affected by barrier length (reported below). Although the longer barrier increased ITIs after either losses or wins, neither the lose-shift or win-stay relationship with ITI was shifted by this procedure (Fig. 3*A*-d). We tested this in two ways: first, qualitatively by computing the coefficient of determination (*r* ^2^) for population data from both the short and long barrier sessions with respect to the one common model fit for all data; and second, quantitatively by computing the difference in the area under the curve (AUC) for each subject across the two barrier conditions. The ITI bins and integration range are held fixed for the AUC computation in each barrier condition for each rat; translation or deformation of the ITI–probability curves induced by the barrier will therefore lead to different integration values, and the difference in AUC between barrier lengths will be nonzero. We computed the difference of AUC for each rat and tested for a nonzero population mean as a test for an effect of the treatment. For lose-shift, population data from each session fit the common model well (*r* ^2^_short_ = 0.82; *r* ^2^_long_ = 0.68, df = 18), and there was no change in the mean difference of the AUC (*t* test that mean difference is 0: *t*(16) = 0.09, *p* = 0.93^s^; Fig. 3*B* inset). Likewise for win-stay, session population data from each condition fit the common model well (*r* ^2^_short_ = 0.69, *r* ^2^_long_ = 0.60, df = 18), and the mean difference of area under the curve across subjects was not different from zero (*t*(14) = 0.55, *p* = 0.59^t^; Fig. 3*D* inset). Because the curves are invariant to increases in ITIs, these data support the hypothesis that the response phenomenon is a result of a within-subject factor such as a decaying memory trace rather than a population effect of motivation or movement speed.

The lose-shift probability decreases in sessions with longer barriers (paired *t* test: *t*(18) = 4.7, *p* = 2E–4^u^; Fig. 3*E*), and this change is accurately predicted by the increase in ITI (Fig. 3*F*) using the log-linear model for each animal (*t* test that mean change in lose-shift is the same as the model prediction: *t*(18) = 0.14, *p* = 0.89^v^; Fig. 3*G*). In other words, the model is able to predict the change in lose-shift based on the change in median ITI for each rat. The overall probability of win-stay does not change (Fig. 3*H*), which is expected because the change in ITI after wins is small with respect to the curvature of the win-stay relationship with ITI. The percentage of rewarded trials is higher in the sessions with the long barriers, as is expected because responses are less predictable when lose-shift responding decreases toward chance level (paired *t* test that mean lose-shift is not increased: *t*(18) =2.45, *p* = 0.02^w^; Fig. 3*I*). Thus, the log-linear model accounts for several features of responding, providing strong evidence that it is an appropriate representation of the relationship between lose-shift responding and ITI.

We next investigated whether the barrier length affects within-session correlations, to additionally assess whether the changes could be due to changes in motivation or outcome valuation. The prevalence of lose-shift responding did not vary within the session, partly because of the high probability of lose-shift in the first few trials in the long barrier condition (main within-subject effect of trial RM-ANOVA: *F*(6,109) = 1.6, *p* = 0.16^x^; Fig. 3*J*). The general trend, however, appears to be increasing lose-shift responding as the session progresses, consistent with the analysis in the previous section (Fig. 2). Also consistent with this previous analysis, the post-loss ITI decreases (RM-ANOVA: *F*(6,109) = 5.7, *p* = 3E–5^y^ ; Fig. 3*K*), and anticipatory licking decreases (RM-ANOVA: *F*(6,108) = 6.8, *p* = 4E–6^z^; Fig. 3*L*) as sessions progress. Note that we used all trials in the computation of anticipatory licking to increase samples, whereas we exclude trials after sampling of both feeders for the other metrics (see Methods). The longer barriers evoked a reduction of lose-shift responding (main within-subject effect of length RM-ANOVA: *F*(1,18) = 8.3, *p* = 0.01^aa^) and increase in ITI (RM-ANOVA: *F*(1,18) = 28, *p* = 5E–5^ab^) but evoked no change in licking (RM-ANOVA: *F*(1,18) = 0.5, *p* = 0.52^ac^) across the session. These data thus support our prediction that motivation decreases within sessions, and that the increased within-session lose-shift prevalence is driven by decreases in ITI after losses. Moreover, lose-shift is again moderately correlated with anticipatory licking in the shorter hallway condition (*r* ^2^ = 0.67; *F*(5) = 10.1, *p* = 0.02^ad^; Fig. 3*L* inset), but data collected in the long barrier condition do not fall on the same line. This indicates that some other factor (e.g., ITI) is needed to predict the relationship between them. This is in stark contrast to the single log-linear relationship between ITI and lose-shift that accounts for data from both barrier conditions (Fig. 3*B*).

In sum, the data in this section provide very strong evidence that lose-shift responding decreases with increased barrier length not because the underlying mechanism changes, but rather because the distribution of the ITI shifts to the right (larger values) so that the memory trace has more time to decay. This indicates that the form of the memory mechanism underlying lose-shift responding is invariant to the animals’ movement speed, and the model can be used to predict changes in lose-shift responding based on changes in ITI.

### Lose-shift responding in the task is inconsistent with reinforcement learning

**Figure 4.**
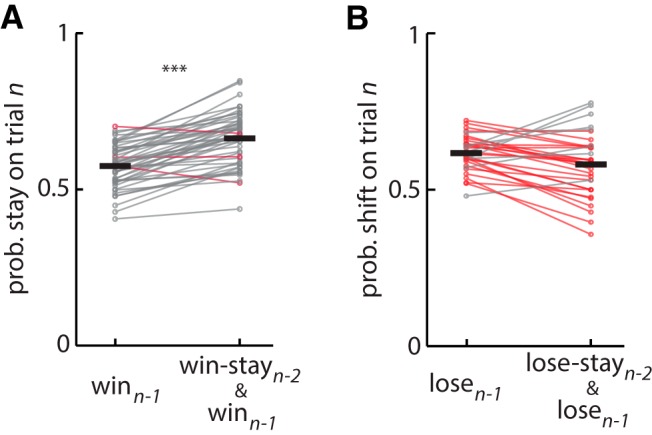
Effect of consecutive wins or losses on choice: test for reinforcement learning. ***A***, Plot of probability of a stay response on trial *n*, after a win (i.e., win-stay; left) or win-stay-win sequence (right) for each rat. The latter is the probability that the rat will chose the same feeder in three consecutive trials given wins on the first two of the set. The data show an increased probability of repeating the choice given two previous wins on the same feeder compared with a win on the previous trial, consistent with RL theory. ***B***, Plot of probability of a switch response on trial *n* after a loss (lose-shift; left) or after a lose-stay-lose sequence (right). The probability of shifting after two consecutive losses to the same feeder is not greater than the probability of shifting after a loss on the previous trial, which is inconsistent with the predictions of RL theory. In both plots, gray lines indicate a within-subject increase in probability, whereas red lines indicate a decrease. ***Statistical significance of increased probability (*p* < 0.001) within subjects.

We have previously shown that the addition of explicit terms for lose-shift and win-stay to a standard reinforcement learning (RL) model improves the prediction of rat choice behavior on this task (Skelin et al., 2014). Moreover, RL does not provide a normative account of the rapid decay of lose-shift responding. Nonetheless, RL mechanisms may contribute to win-stay or lose-shift responding. For instance, a large learning rate will cause choice to be highly sensitive to the previous trial by driving large increases (decreases) of the choice after wins (losses). We thus tested a fundamental prediction of RL: successive wins or successive losses on the same choice should have an additive (albeit sublinear) effect on choice. For instance, the probability of a stay response after a win-stay-win sequence on the same feeder should be greater than that after a win irrespective of outcomes in the past. Formally, this is expressed by the inequality: $\text{Prob}\left(stay_{n}|win_{n-1},stay_{n-1},win_{n-2}\right)>\text{Prob}\left(stay_{n}|win_{n-1}\right)$. Indeed, we find that the probability of staying after a win-stay-win sequence is greater than the probability of staying after a win (paired *t* test that the above equality is not true for rats with at least 25 samples of win-stay-win sequences: *t*(48) = 10.2, *p* = 1E–13^ae^; Fig. 4*A*). Likewise, the probability of switching should be increased after a lose-stay-lose sequence, formalized by $\text{Prob}\left(shift_{n}|lose_{n-1},stay_{n-1},lose_{n-2}\right)>\text{Prob}\left(shift_{n}|lose_{n-1}\right)$. However, we find that this is not the case (paired *t* test that the above equality is not true for all rats with at least 25 samples of lose-stay-lose sequences: *t*(32) = 2.2; *p* = 0.99^af^; Fig. 4*B*). Thus, the probability of shifting is not increased after two consecutive losses at one feeder compared with the probability of shifting after loss on the previous trial, which is inconsistent with the foundational concept of RL that the value of the feeder should be additionally decremented by the second loss, and therefore the likelihood of choosing the other feeder should be higher (e.g., shift). In sum, the RL concept of reinforcement-driven value learning is consistent with responding after wins, but not after losses. This suggests that the neural mechanisms involved in lose-shift are distinct from those involved in RL. Conventional RL has the facility to implement win-stay-lose-shift, although not to the extent evident in the present data. To evaluate the predictive power of a standard RL algorithm (Q-learning) compared with a pure win-stay/lose-shift strategy, we computed the prediction accuracy for each model on one session from each rat in a cohort (see Methods, *n* = 19). The win-stay/lose-shift correctly predicted 60 ± 1% of responses, whereas Q-learning predicted 52 ± 1% of responses. It is worth noting that we tested the prediction of Q-learning on the same data that was used to fit the model parameters so as to produce the highest possible accuracy regardless of overfitting. Nonetheless, these data provide strong evidence that the win-stay/lose-shift strategy better accounts for responding on this task (*t* test of mean prediction accuracy between models: *t*(34) = 5.2, *p* = 1E–5^ag^). It is unsurprising that RL does not account for responses on this particular task because the expected long-term utility of both feeders is equivalent. If the probability or amount of reward were unequal at the two feeders, the brain would likely engage RL systems to overshadow the lose-shift mechanisms presenting here.

### Lose-shift responding is stationary during training, whereas win-stay is acquired

**Figure 5.**
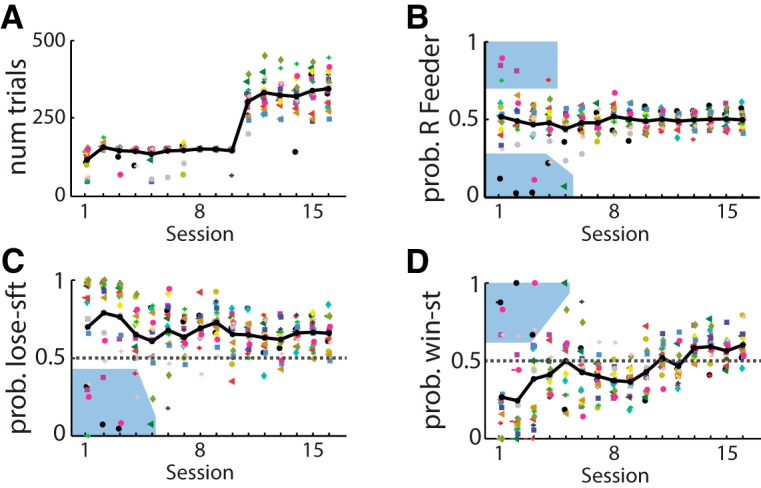
Responses during every training session for one cohort. Responses plotted for each rat (symbol-color) and each day of training. Session 1 is the second time the rats were placed into the behavioral box, and reward probability was *p* = 0.5 for each feeder regardless of previous responses or rewards. ***A***, Number of trials completed in each session. Rats were allowed 90 min to complete up to 150 trials in sessions 1–10, and hallways of increasing lengths were introduced in sessions 3–8. ***B–D***, Plot of the probability of responding to the rightward feeder, probability of lose-shift, and probability of win-stay during the first 16 sessions. The majority of rats showed no side bias, strong lose-shift, and very little win-stay in initial trials. Only a few rats showed initial side bias, and therefore little lose-shift and strong win-stay (blue shading in panels ***B–D***). Lose-shift was invariant over training, whereas win-stay increased (see text). Dark lines indicate median across all subjects for each day.

We next sought to determine whether the prevalence of lose-shift responding is related to aspects of the task, such as the competitive algorithm or barriers, which are atypical of other tasks. We therefore examined the probability of lose-shift and win-stay in a new cohort of rats (*n* = 17) undergoing a modified training schedule. In attempt to normalize learning across subjects, rats were allowed 90 min in the behavioral box to perform up to a maximum of 150 trials per session over the first 10 days, and then unlimited trials for 90 min in subsequent sessions (Fig. 5*A*). Increasingly longer barriers were introduced in sessions 3–8. A few rats initially had a strong side bias (blue shaded region in Fig. 5*B*), and consequently tended to stay regardless of loses or wins (blue shaded region in Fig. 5*C*, *D*). The majority of rats, on the other hand, showed prominent lose-shift responding across all sessions, even during the second session in the apparatus in which the competitive algorithm was not used and the probability of reward was *p* = 0.5 regardless of previous choices. Nonetheless, the probability of lose-shift (median = 0.86, including the animals with side bias) was significantly higher than chance on this session (two-sided Wilcoxon signed-rank test for median = 0.5, *n* = 17, *p* = 0.03^ah^). Moreover, the probability of lose-shift in the population did not vary across the training sessions (within-subjects main effect of session RM-ANOVA: *F*(15,150) = 0.54, *p* = 0.91^ai^; Fig. 5*C*). In contrast, the probability of win-stay was initially less than chance (Wilcoxon; *n* = 17, *p* = 0.01^aj^) and increased across testing sessions (RM-ANOVA: *F*(15,150) = 2.3, *p* = 5E–3^ak^; Fig. 5*D*). These features of the data would be even stronger by omitting the sessions in which rats had a large side bias. These data reveal that lose-shift responding is prevalent across all sessions, whereas win-stay is acquired during training, again supporting the hypotheses that they are mediated by separate processes. The pronounced lose-shift responding in the first several sessions indicates that it is not the barriers or competitive algorithm that induces animals to utilize this response strategy.

## Discussion

The present data show that reward omission has a pronounced short-lasting effect on subsequent choice, which can be described by the classic notion of lose-shift responding that decays over several seconds. Several features of rodent lose-shift are distinct from those of win-stay: (a) their probability is not positively correlated among subjects; (b) their temporal dependence on ITI is dissimilar; and (c) lose-shift is prevalent from the first day of training and does not diminish, whereas win-stay is acquired during training. These data provide further evidence that win-stay and lose-shift are mediated by dissociated neural mechanisms. The temporal dependence of lose-shift responding presents a confound for the study of choice in rodents and other animals that likely influences performance in the many operant choice tasks with short ITIs. Moreover, manipulations that affect ITI (e.g., drugs, stress) will alter the prevalence of lose-shift and win-stay responses. Studies that do not control for this are difficult to interpret because tasks solvable by lose-shift will be facilitated by reduced ITI independently of other putative mechanisms. Lose-shift responding is thus an important latent variable to consider in behavioral studies of choice.

The highly prominent lose-shift responding over the 7-s interval considered here is not explained by conventional RL theory. In particular, the rapid decay of switching probability during the ITI has no normative basis in RL. Furthermore, that the probability of shifting after a lose-stay-lose sequence is not greater than that of shifting after a single loss is counter to the fundamental prediction of RL that subsequent losses on the same feeder should decrement the value of the action and therefore increase the probability of switching. On the other hand, the properties of win-stay are more consistent with RL, in that consecutive wins do increase the probability of a stay response. The dependence of win-stay on ITI, however, remains unexpected. This dissociation is counter to conventional RL formulations, in which wins and losses influence choice by modulating a singular value attached to actions or outcome states (Watkins and Dayan, 1992; Sutton and Barto, 1998). We instead propose that the lose-shift phenomenon can be characterized as an intrinsic choice reflex because of its prevalence in the task (despite being a non-optimal solution), its failure to diminish over thousands of trials, its reliable time course, and its apparent independence of neural systems involved in executive functions (Skelin et al., 2014).

The brief lose-shift system involving the sensorimotor system studied here is dissociated from the reinforcement learning signals in ventral striatum and orbitofrontal cortex observed in many other studies (Samejima et al., 2005; Daw et al., 2006; Paton et al., 2006; Matsumoto et al., 2007; Schönberg et al., 2007; Hori et al., 2009; Ito and Doya, 2009; Bromberg-Martin et al., 2010b; Gan et al., 2010; Alexander and Brown, 2011; Day et al., 2011). We argue that lose-shift is an adjunct to RL in the guidance of choice; the neural mechanisms for RL likely solve problems requiring processing of value or utility over many trials to establish responding rates to various choice options, whereas the lose-shift mechanism likely introduces exploration among the choice only on a trial-by-trial scale. The behavioral purpose of its 7- to 8-s time course is unclear, but this temporal window is supported by some of the few other reports that provide relevant evidence. Direct optogenetic activation of D2DR-expressing striatal cells in the dorsal striatum of mice results in place avoidance for about 10 s (Kravitz et al., 2012), and the behavioral effect of losses during a lever-pressing task is observed only when ITIs are less than ∼15 s (Williams, 1991). Moreover, win-stay/lose-shift behavior is prominent in pigeons only when ITIs are <10 s (Rayburn-Reeves et al., 2013). The emergence of rapidly decaying lose-shift behavior across species and tasks, even when it is not needed or is suboptimal, suggests it is a general feature of choice intrinsic to its underlying mechanisms.

The memory trace supporting lose-shift is only one of several memory systems in the brain. Rats can maintain information related to reinforcement over much longer intervals, and performance on these longer-interval tasks is often sensitive to disruption of the prefrontal cortex (Euston et al., 2012). This suggests that goal-directed behavioral control involving prefrontal cortex has a longer memory frame than the one considered here. We speculate that this difference in time frame accounts for the discrepancy of our results from that of devaluations experiments, which indicate that behavior mediated by sensorimotor striatum is not sensitive to changes in the affective value of reinforcements (Yin et al., 2004; Quinn et al., 2013). This result has had a profound influence on many current theories of choice (Daw et al., 2005; Balleine and O’Doherty, 2010; Gruber and McDonald, 2012; van der Meer et al., 2012). In devaluation, the affective state of the animal is altered with either satiation or illness paired with the outcome, and the memory time is hours to days. Many regions of the prefrontal cortex and subcortical limbic structures encode affective information over time periods spanning minutes to months (Euston et al., 2012) and project heavily to the medial and ventral striatum (McGeorge and Faull, 1989; Vertes, 2004; Voorn et al., 2004). It is not surprising, then, that devaluation depends on medial striatum and not dorsolateral striatum. The lose-shift phenomenon studied here occurs over several seconds, and reward omission likely does not elicit a strong affective component because rats are denied only a small amount reward on each trial relative to the total reward intake over the session. We therefore propose that the inverse role of the dorsolateral striatum in devaluation and lose-shift behaviors derives from the differences in memory time interval and sensory domain (affective vs. sensory). In other words, the sensorimotor systems have explicit access to recent sensory information (including that related to reward) needed for lose-shift, but not direct access to remote affective information as needed for devaluation effects.

The percent of rewarded trials in our sample is on par with that of rats (Tervo et al., 2014) and nonhuman primates (Lee et al., 2004) competing against the same algorithm, albeit with different motoric demands. The probability of lose-shift alone is not reported in either study, but the non-human primates show only a slight amount of win-stay/lose-switch (Prob = 0.53–0.57) in the competitive task, similar to humans (Prob = 0.54–0.57; Hu et al., 2010). We speculate that the primate prefrontal cortex normally suppresses lose-shift by the sensorimotor striatum, so that primates lose-shift less than rats. Rats appear to strongly use sensorimotor systems to respond during the task, and therefore exhibit high amounts of lose-shift throughout training and testing.

Lose-shift responding is suboptimal in the present task, but its persistence is not likely to be an artifact of the task design. Lose-shift responding is prevalent on the second day of training without barriers and without the competitive algorithm, and is invariant across training and testing. Other experiments have also revealed that rats do not perform optimally on binary choice tasks with dynamic reinforcements (Sul et al., 2011). Last, lose-shift is pervasive across many species and tasks (Mishkin et al., 1962; Schusterman, 1962; Olton et al., 1978; Evenden and Robbins, 1984; Means and Fernandez, 1992; Komischke et al., 2002; Lee et al., 2004; Frank et al., 2007; Amodeo et al., 2012; Rayburn-Reeves et al., 2013; Skelin et al., 2014; Wang et al., 2014), and those abovementioned studies that report timing effects are consistent with the decay in our data. In sum, several lines of indirect evidence indicate that the lose-shift phenomenon studied here is not unique to the task, but rather appears to be a default strategy in many situations, and is therefore relevant to many other behavioral tests with short ITIs.

The properties of lose-shift revealed here suggest it is an intrinsic feature of neural choice mechanisms in the striatum that can be described as a choice reflex; it is unlearned, prevalent in multiple cohorts, persistent, has a reliable time course, and involves the sensorimotor striatum. As such, the addition of explicit terms in RL models that include these properties will likely continue to improve model fits to data, particularly in tasks with short ITI and sensorimotor solutions (Ito and Doya, 2009; Rutledge et al., 2009; Skelin et al., 2014).

In conclusion, lose-shift responding plays a simple but important role in trial-by-trial choice adaptation in some situations, particularly those with repetitious actions and rapid trials, and appears to work in parallel with reinforcement learning and other control mechanisms in dissociated neural structures to guide choice. Our data provide further evidence that theories of sensorimotor striatum function related to choice behavior must expand from the current focus on gradual sensory-response associations and habit formation (Jog et al., 1999; Daw et al., 2005; Balleine and O’Doherty, 2010; Devan et al., 2011; Gruber and McDonald, 2012; van der Meer et al., 2012) to also include rapid response adaptation that is dependent on a decaying memory trace.

## Synthesis

Decisions are customarily a result of the Reviewing Editor and the peer reviewers coming together and discussing their recommendations until a consensus is reached. When revisions are invited, a fact-based synthesis statement explaining their decision and outlining what is needed to prepare a revision will be listed below. The following reviewer(s) agreed to reveal their identity: Michael Frank, Norman White. Note: If this manuscript was transferred from JNeurosci and a decision was made to accept the manuscript without peer review, a brief statement to this effect will instead be what is listed below.

This is an interesting extension of the authors' previous paper (Skelin, et al., The European Journal of Neuroscience, 39, 1655, 2014) that unexpectedly showed that these behaviors were impaired by lesions of the dorsal striatum. This paper analyzes data from normal rats to illustrate that the tendency to shift behavioral responding after reward omission ("lose-shift") is subject to decay as a function of time between trials, which has not been documented in this way before. This work is important because it's approach to understanding these behaviors is unique, because it expands our knowledge of the functions of the striatum, and because it has implications for the design of experiments in which the failure to account for these short term behavioral tendencies could lead to serious errors of interpretation.

Major Comments

1a)Trials were self-paced so the ITI was computed as time from feedback to next nose poke (rats) to initiate next trial. But then the delay time is confounded with motivation/satiation which could explain differences in win/stay vs lose-shift instead of, or in addition, to the delay (memory trace) itself. The authors address this by introducing barriers to affect ITI "without affecting motivation", but the barrier itself introduces differential physical effort which also enters into motivation. Thus what the authors attribute to delay with longer ITI's could also relate to increased effort and thus lower choice value. While I am not aware of a theory that states lower choice values should translate into reduced lose-shift, one could imagine that this might obtain from simply more random behavior as values go down, which would be predicted from standard softmax functions in RL, and this point could be acknowledged as a limitation.

1b)Increasing the length of the barriers would have increased the ITI as the authors say, but would also have increased the cost of each feeder choice and the time taken to reach the feeders after a port entry. The possible influence of these factors on the rats' behavior is not mentioned, but should be.

2)I wasn't convinced by the analysis of lose-stay-lose, where 2 losses in a row would predict higher chance of shifting than a single loss. This is because the tendency to stay is itself a behavioral state (irrespective of outcome) and any time an animal repeats the same action (stay) they are more likely to be in that state and hence to repeat it again, regardless of win or loss. While that in itself is inconsistent with RL alone, several studies that show this behavioral perseveration ("sticky choice") tendency rides on top of RL processes (e.g., Schonberg et al 2007 Jneurosci; Lau and Glimcher 2005), rather than challenges their existence altogether. The authors could either acknowledge this or amend their Q learning algorithm to include the sticky choice component and determine if it is affected by ITI

3)Rats learned to poke their noses into a port when a signal light came on, and then obtain a drop of a sucrose solution from one of two feeders located on either side of the port. The feeder that delivered the sucrose on any given trial was determined according to an algorithm that is not fully explained in the paper. It apparently used information from the each rat's behavior over the entire session and from the last few trials to minimize the number of sucrose deliveries following rewarded and unrewarded trials. The authors say that the optimal strategy was to choose randomly. We are left to suppose this means that any deviation from random performance implies some kind of learning process. The authors should specify what they mean by these findings.

Specific comments:

4)The title and some parts of the discussion still refer to human experiments (the previous manuscript for JN included a human experiment but this was omitted from the current manuscript so the text should be adjusted).

5) The authors say this is a self-paced task, but the 2 signal lights located on the wall above the port were illuminated to signal the start of a trial, after which the rats had 15 sec to nose-poke the port and contact one of the feeders. We are not told what happened if they failed to meet this restriction. More importantly, although we are told that the signal lights turned off when a rat contacted a feeder, we are not told if or when they were turned back on. It seems obvious that these signal lights could have acquired conditioned stimulus properties that would have influenced the rats' behavior if and when they were illuminated during the ITI. I think this issue needs to be addressed. (Some of this information about the lights is in the Apparatus section, some is in the Behavioral Task section and some is in the Results section. Surely it should all be in the Behavioral Task section?)

6) The fact that maximum probability of a win-stay response occurs later than the maximum probability of a lose-shift response is described as a post-reinforcement pause. But the Felton and Lyon article cited (and other articles on this phenomenon) describes post-reinforcement pauses of 120-7000 sec observed in 4 pigeons on fixed ratio schedules of 25 - 150 responses per access to food. Can the authors really mean to suggest that the 3-4 sec increase in the mean latency for responses following win-stay over lose shift in this experiment is due to the same process? This really seems unlikely to me. What does seem likely is that the increase is probably at least partly due to the time required to swallow the sucrose solution after a win. Another phenomenon that could account for the short ITI after a loss is the invigorated performance that often follows failure to obtain expected food or water, called frustrative non-reward in the old literature (Amsel, Psychological Bulletin, 55, 102, 1958). There's at least a PhD thesis in the investigation of the contribution to this short-term invigoration phenomenon of the increase in striatal dopamine release caused by omission of an expected reward.

7) As I understand it, the section on the distinction between a "population component" and trace decay as explanations for the linear decline in probability of a lose-shift response comes down to whether it is a within or between subject phenomenon. This did not become clear to me until lines 221-2, *after* the analysis was described. I did not understand the implication of the term "population component" at the beginning of the section. I suggest giving the within-between explanation at the start of the section.

8) When rats were observed from the start of training win-stay responding was low at first, and increased gradually over trials, consistent with the idea that the response is gradually increased each time it is reinforced. However, lose-shift probability was above chance on the second trial and remained there at a steady level over the 16 training sessions. I agree with the authors' interpretation of this finding as suggesting that lose-shift is a kind of innate behavioral tendency. It might strengthen their argument to mention that this kind of lose-shift behavior has been observed in many situations, such as the 8-arm radial maze (Olton and Schlosberg, Journal of Comparative and Physiological Psychology, 92, 609, 1978) and in such tasks as DNMS in monkeys (Mishkin, Prockop and Rosvold, Journal of Comparative and Physiological Psychology, 55, 178, 1962).

9) The basis of the discussion of the relationship between reinforcer devaluation and lose-shift is not really clear to me. Specifically, why is it contradictory that DLS may mediate the very short term lose-shift responding shown here but not mediate reinforcer devaluation? Is it because lose shift is assumed to be based on some kind of aversion? If so, both this assumption (or for the alternative if that's not the reason) and the reasons for it need to be made clear up front. I think the authors are right about the reasons for this apparent contradiction: both the short-term win-stay and lose-shift are based on working memory that uses (sensory) information about the learning situation, but is not influenced by any affective responses that may be induced in the situation. One simple reason for this could be that the time scale over which affective responses occur, are perceived, and influence behavior may be longer than the ITIs in this experiment.

10) Finally, although there are no lesions in this paper, it is clearly an extension of the authors' previous demonstration (Skelin, et al., The European Journal of Neuroscience, 39, 1655, 2014) that striatal lesions impair both win-stay and lose-shift responding. These behaviors seem clearly incompatible with the S-R learning that has been attributed to the dorsolateral striatum, but they could be based on A-O, learning attributed to the dorsomedial striatum and, possibly the patch compartment that is prominent in that part of the striatum. As the authors recognize, the short-term memories studied here are within the range of those addressed by the working memory literature. There is evidence for the involvement in working memory of the patch compartment in conjunction with the pre-frontal cortex (e.g., Moustafa and Maida, Cognitive Systems Research, 8, 262, 2007; Brown, et al., The Journal of Neuroscience, 19, 10502, 1999). Conventional lesions of the medial and lateral dorsal striatum produce very non-specific damage to these compartments, making it very difficult to distinguish their functions. There are methods for selectively ablating the compartments, but until they are used in behavioral studies the data on basal ganglia function will probably remain confusing.

11) The authors acknowledge several people for help with histology and catheter implantation, neither of which is included in this manuscript. Please modify the acknowledgements.
